# Microstructural and functional alterations of the ventral pallidum are associated with levodopa‐induced dyskinesia in Parkinson's disease

**DOI:** 10.1111/ene.16147

**Published:** 2023-11-17

**Authors:** Yawen Gan, Dongning Su, Zhe Zhang, Zhijin Zhang, Rui Yan, Zhu Liu, Zhan Wang, Junhong Zhou, Joyce S. T. Lam, Tao Wu, Jing Jing, Tao Feng

**Affiliations:** ^1^ Department of Neurology, Beijing Tiantan Hospital Capital Medical University Beijing China; ^2^ China National Clinical Research Center for Neurological Diseases Beijing China; ^3^ Tiantan Neuroimaging Center of Excellence, Beijing Tiantan Hospital Capital Medical University Beijing China; ^4^ Hinda and Arthur Marcus Institute for Aging Research Hebrew SeniorLife Roslindale Massachusetts USA; ^5^ Harvard Medical School Boston Massachusetts USA; ^6^ Pacific Parkinson's Research Centre, Djavad Mowafaghian Centre for Brain Health University of British Columbia Vancouver British Columbia Canada

**Keywords:** levodopa‐induced dyskinesia, MRI, multimodal, Parkinson's disease, ventral pallidum

## Abstract

**Background and purpose:**

The ventral pallidum (VP) regulates involuntary movements, but it is unclear whether the VP regulates the abnormal involuntary movements in Parkinson's disease (PD) patients who have levodopa‐induced dyskinesia (LID). To further understand the role of the VP in PD patients with LID (PD‐LID), we explored the structural and functional characteristics of the VP in such patients using multimodal magnetic resonance imaging (MRI).

**Methods:**

Thirty‐one PD‐LID patients, 39 PD patients without LID (PD‐nLID), and 28 healthy controls (HCs) underwent T1‐weighted MRI, quantitative susceptibility mapping, multi‐shell diffusion MRI, and resting‐state functional MRI (rs‐fMRI). Different measures characterizing the VP were obtained using a region‐of‐interest‐based approach.

**Results:**

The left VP in the PD‐LID group showed significantly higher intracellular volume fraction (ICVF) and isotropic volume fraction (IsoVF) compared with the PD‐nLID and HC groups. Rs‐MRI revealed that, compared with the PD‐nLID group, the PD‐LID group in the medication ‘off’ state had higher functional connectivity (FC) between the left VP and the left anterior caudate, left middle frontal gyrus and left precentral gyrus, as well as between the right VP and the right posterior ventral putamen and right mediodorsal thalamus. In addition, the ICVF values of the left VP, the FC between the left VP and the left anterior caudate and left middle frontal gyrus were positively correlated with Unified Dyskinesia Rating Scale scores.

**Conclusion:**

Our multimodal imaging findings show that the microstructural changes of the VP (i.e., the higher ICVF and IsoVF) and the functional change in the ventral striatum–VP–mediodorsal thalamus–cortex network may be associated with pathophysiological mechanisms of PD‐LID.

## INTRODUCTION

Levodopa‐induced dyskinesia (LID) refers to various abnormal involuntary movements that are often observed in Parkinson's disease (PD) patients after prolonged levodopa treatment. It is a debilitating symptom that can manifest in discrete body parts or can become generalized, and severely reduces the quality of life of those affected [1]. Approximately 90% of PD patients experience LID after 10 years of levodopa treatment [2]. Despite its prevalence, the neural mechanism underlying PD with LID (PD‐LID) remains poorly understood. Current evidence suggests there could be an imbalance in the direct and indirect pathways of the cortico‐basal ganglia–thalamo‐cortical circuit in PD‐LID [3]. For example, one positron emission tomography (PET) study found that PD‐LID patients had a higher dopamine turnover in the putamen than patients who had PD without LID (PD‐nLID) [4]. The altered signals originating from the putamen might inhibit the indirect pathway and stimulate the direct pathway. However, studies in 1‐methyl‐4‐phenyl‐1,2,3,6‐tetrahydropyridine‐treated primates have shown that the activity of the indirect pathway is not diminished in dyskinetic animals [5, 6], suggesting that the cause of PD‐LID cannot be fully attributed to the classic circuit and that other nuclei might also be involved in the basal ganglia–cortical circuit.

The ventral pallidum (VP) is a major component of the ventral basal ganglia and is involved in a variety of functions, such as movements, reward, memory and learning [7, 8]. Previous studies have shown that the VP receives afferents from the ventral striatum, including the ventral caudate and putamen [9] and nucleus accumbens [10], and projects to the mediodorsal thalamus [11] and subsequently to the frontal cortex [12]. The frontal cortex projects back to the ventral striatum [13], together forming a circuit regulating motivation and locomotion [14]. Evidently, the VP plays a pivotal role in the basal ganglia–cortical motor pathway. In addition, earlier studies demonstrated that the injection of γ‐aminobutyric acid (GABA) receptor antagonists within the VP could induce involuntary movements including sniffing, gnawing, tongue protrusion, and chewing behaviours in rats and cats [15], suggesting that the VP contributes to abnormal movements. However, it is still not known if these VP‐mediated abnormal movements are involved in the development of PD‐LID.

Recently, advanced neuroimaging techniques have shown potential in identifying the structural and functional alterations of the brain regions associated with PD‐LID. Several structural and resting‐state functional magnetic resonance imaging (rs‐fMRI) studies have demonstrated the role of an anomalous brain functional network in PD‐LID involving the inferior frontal gyrus [16], pre‐supplementary motor area, and putamen [17]. A recent study found that patients with PD who were vulnerable to LID had significant inward deformation in the thalamus, especially in the ventral anterior and mediodorsal nuclei, as compared to those resistant to LID [18]. These results highlight the role of the frontal gyrus, putamen, and mediodorsal thalamus of the cortico‐basal ganglia–thalamo‐cortical circuit in PD‐LID. As part of this circuit, the VP is associated with relaying information to the motor cortices [19]; thus, structural and functional changes of VP may play a role in PD‐LID. Multimodal MRI techniques, such as T1‐weighted, diffusion MRI (dMRI) and functional MRI (fMRI), can reflect the macrostructural and microstructural integrity of grey matter [20, 21] as well as the functional activity in different brain networks [22], which could be used to better characterize the alterations in the VP and help elucidate the role of the VP in the pathophysiology of PD‐LID.

In this study, we explored the alterations of the VP in PD‐LID using multimodal MRI, including T1‐weighted MRI, quantitative susceptibility mapping (QSM), multi‐shell dMRI, and fMRI. We compared the brain grey matter density, iron accumulation, neurite microstructure of the VP, and functional connectivity (FC) between the VP and other brain regions among PD‐LID patients, PD‐nLID patients, and healthy controls (HCs). The aim of this study was to provide novel and comprehensive insights into the VP with regard to the pathophysiology of LID, which may yield a potential new target for neuromodulation in the treatment of LID.

## MATERIALS AND METHODS

### Participants

Seventy PD patients and 28 HCs were enrolled in this study. PD participants were categorized into a PD‐LID group (*n* = 31) and a PD‐nLID group (*n* = 39) based on the diagnosis of LID. LID was diagnosed by two experienced movement disorder specialists, through a detailed interview and review of medical records, and with a score of greater than 1 on the Movement Disorder Society‐Unified Parkinson's Disease Rating Scale (MDS‐UPDRS) items 4.1 and 4.2 during the medication ‘on’ state. The inclusion criteria for PD patients were as follows: (i) clinical diagnosis of PD based on the Movement Disorder Society Clinical Diagnostic Criteria for Parkinson's Disease; (ii) a minimum of half‐year duration of levodopa therapy; and (iii) presence or absence of LID following an acute levodopa test. The exclusion criteria were: (i) contraindications to MRI (e.g., claustrophobia or MRI‐incompatible implants in the body); (ii) presence of vascular brain lesions, brain tumour, or marked brain atrophy on MRI scan; (iii) intake of sedative and hypnotic medications; (iv) diagnosis of atypical parkinsonism; and (v) excessive movement artifacts.

Before the MRI scans, PD patients underwent clinical assessments in the medication ‘off’ state that included Hoehn and Yahr (H&Y) staging, MDS‐UPDRS Part III score, Mini‐Mental State Examination (MMSE) score, Montreal Cognitive Assessment (MoCA) score and levodopa equivalent daily dose (LEDD) calculation [23]. In the medication ‘on’ state, the Unified Dyskinesia Rating Scale (UDysRS) for severity of dyskinesia was assessed in PD‐LID patients [24].

The MRI scans were performed after at least 8 h of withdrawal from anti‐parkinsonian drugs for all PD participants. In addition, 28 age‐ and gender‐matched HCs without a history of neurological or psychiatric conditions or abnormal brain MRI were recruited. The study received approval from the Ethics Committee of Beijing Tiantan Hospital, according to the Helsinki Declaration. All participants provided written informed consent.

### MRI data acquisition

All study participants were scanned on a 3T‐MRI scanner (MAGNETOM Prisma, Siemens Healthcare, Erlangen, Germany) using a 64‐channel head/neck coil. Three‐dimensional T1‐weighted MRI, three‐dimensional QSM, multi‐shell dMRI, and fMRI were performed in all participants. The detailed methods of MRI data acquisition are included in Appendix S1.

### Image analysis

**FIGURE 1:**
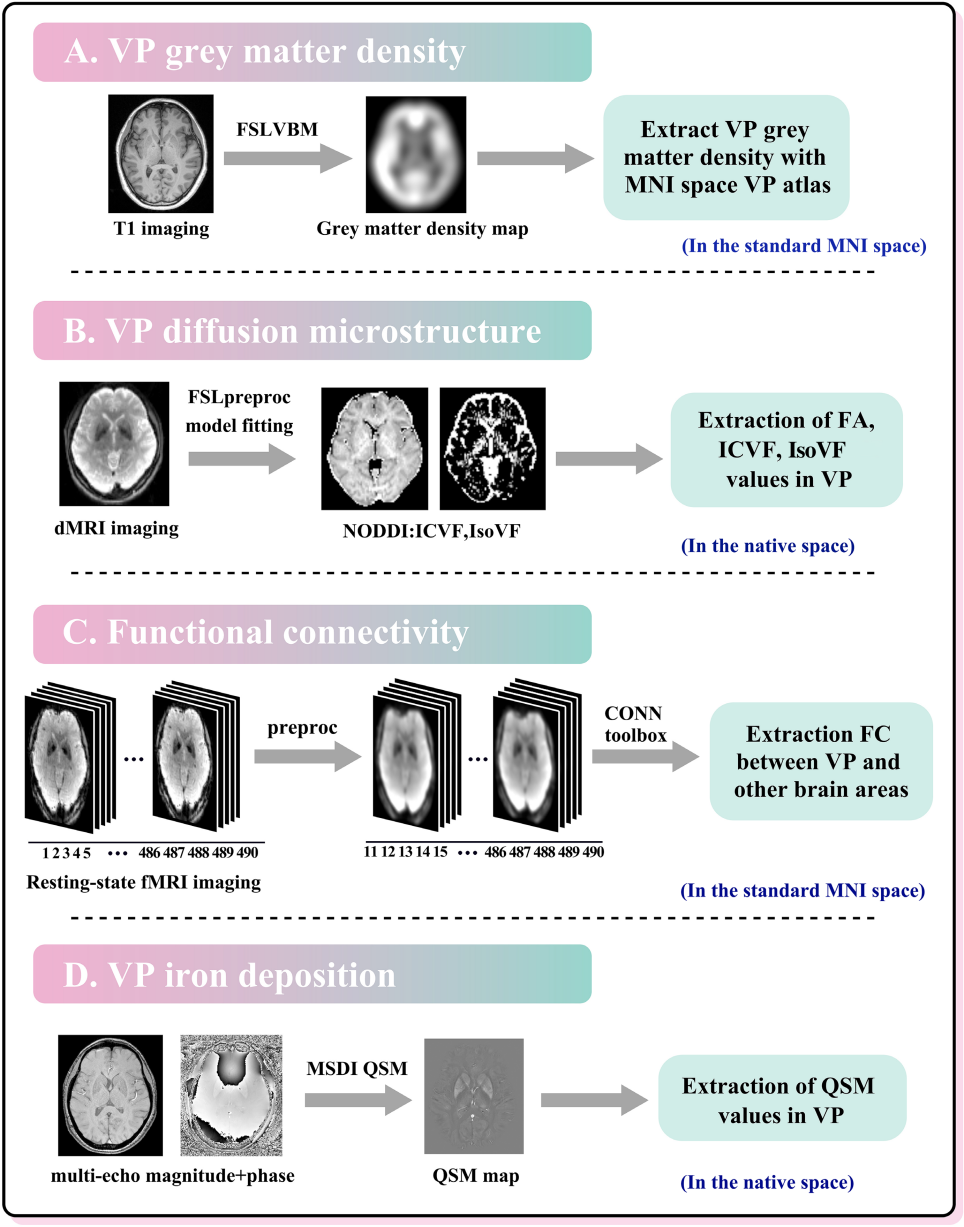
Flowchart of the imaging analysis steps. dMRI, diffusion magnetic resonance imaging; fMRI, functional magnetic resonance imaging; FA, fractional anisotropy; FC, functional connectivity; FSLVBM, FMRIB Software Library voxel‐based morphometry; ICVF, intracellular volume fraction; IsoVF, isotropic volume fraction; MNI, Montreal Neurological Institute; MSDI, multi‐scale dipole inversion; NODDI, neurite orientation dispersion and density imaging; QSM, quantitative susceptibility mapping; VP, ventral pallidum.

The flowchart of the imaging analysis steps used in this multimodal study is illustrated in Figure 1. The regions of interest (ROIs) from T1‐weighted MRI using the California Institute of Technology (CIT168) atlas [25] were co‐registered to different MRI modalities including QSM, dMRI and rs‐fMRI (to the distortion‐corrected *b* = 0 image in dMRI and the magnitude image in QSM). Specifically, the nonlinear transformation between the T1‐weighted MRI and the Montreal Neurological Institute (MNI) 152 template was generated and saved. Then, the rigid transformation between T1‐weighted MRI and specific MRI modality was generated. The ROIs (described below) were mapped to the specific modality image space using the combined transformation from MNI152 space to T1 (nonlinear) and T1 to specific modality image (rigid). For imaging modalities including grey matter density and rs‐fMRI, the extraction of ROI data was performed in the standard brain space [26]. While in dMRI and QSM, the extraction of ROI data was performed in the native space to avoid several confounds associated with spatial normalization of ROIs [27, 28, 29]. The registration and ROI generation were conducted in the FMRIB Software Library (FSL) [30].

### Regions of interest for analysis

The VP ROIs were defined using the CIT168 atlas mentioned above. We analysed the grey matter density, QSM values and diffusion metrics of the bilateral VP, and the FC between the bilateral VP and the brain regions in both hemispheres.

### Grey matter density analysis

The grey matter density was calculated with FSL voxel‐based morphometry pipeline using T1‐weighted MRI [31]. First, the brain‐extracted T1 images were segmented into grey matter, white matter and cerebrospinal fluid (CSF). Then, a study‐specific grey matter template was created in two steps. The grey matter images were affine‐registered to the MNI152 template and the resulting images were averaged to create a first‐pass template. Next, the grey matter images were nonlinearly registered to the first‐pass template and then averaged to obtain the final template at 2‐mm resolution in standard space. Finally, the grey matter images were nonlinearly registered to the final template and smoothed with a Gaussian kernel with sigma = 8 mm. The smoothed grey matter image in standard space was used for the grey matter density metric extraction from left and right VP ROIs.

### Diffusion MRI analysis

For diffusion imaging data, field inhomogeneity‐induced distortion, patient motion, and eddy‐current‐related artifacts were corrected using the TOPUP and EDDY toolbox [32] in FSL [30] with the data collected with reversed phase‐encode blips (AP and PA). The diffusion tensor imaging (DTI) metrics, including fractional anisotropy (FA) and mean diffusivity (MD) from the DTI model, and the diffusion microstructure metrics, including intracellular volume fraction (ICVF; termed neurite density index), isotropic volume fraction (IsoVF), and orientation dispersion (OD) from the neurite orientation dispersion and density imaging (NODDI) model [33], were all analysed in the VP ROIs.

### Quantitative susceptibility mapping image analysis

The multi‐echo magnitude and phase images were saved, and QSM (parts per million) was calculated using the QSMbox toolbox's multi‐scale dipole inversion algorithm [34] (available at https://gitlab.com/acostaj/QSMbox). The brain mask was extracted from the T1‐weighted image and registered to the QSM image space. The susceptibility map with CSF as the zero reference was used for QSM ROI statistical analysis.

### Functional MRI image analysis

The fMRI analysis was performed with the Functional Connectivity Toolbox (CONN) [35] (version 18b) and SPM12 (www.fil.ion.ucl.ac.uk/spm/software/spm12/) in MATLAB (version R2020b MathWorks, Natick, MA, USA). The bilateral VP of both hemispheres was selected as seed region. Seed‐to‐seed analysis was conducted to analyse the FC changes between the VP and other brain regions. Based on previous findings, we selected VP‐connected brain regions as other seeds, including the frontal lobe (inferior frontal gyrus, middle frontal gyrus, superior frontal gyrus, precentral gyrus and frontal pole) [14, 36], insular cortex [37], anterior and posterior cingulate cortex, precuneus cortex [38], hippocampus [39], amygdala, ventral tegmental area, substantia nigra pars compacta, striatum (the subregions of caudate and putamen; e.g., anterior and posterior caudate, anterior and posterior ventral putamen, anterior and posterior dorsal putamen), nucleus accumbens shell and core, and mediodorsal thalamus [19, 40]. The resting state connectivity between the VP and other brain regions for each subject was extracted from the CONN toolbox after first‐level analysis [38], and converted into z‐maps using Fisher's r to z transformation to enhance normality.

The detailed processing description of dMRI and fMRI analysis is shown in Appendix S1.

### Statistical analysis

All statistical analyses were performed using IBM SPSS Statistics Version 25.0 and R version 4.1.2. The significant differences in demographics among the three groups were examined using analysis of variance (ANOVA) and the chi‐squared test (for the number of women participants). ANOVA was used to examine the difference in MoCA scores and age within the three groups. A two‐sample *t*‐test was used to compare the difference in MDS‐UPDRS Part III scores in the medication ‘off’ and ‘on’ state, age of onset, response to levodopa, and LEDD between the PD‐LID and PD‐nLID groups. The chi‐squared test was also used to analyse the proportion of patients whose right/left body side was more severely affected in the two PD groups. The Mann–Whitney test was performed to compare disease duration and H&Y stage between the PD‐LID and PD‐nLID groups as their demographics were not normally distributed. One‐way ANOVA, followed by Bonferroni's post hoc analysis, was used to compare grey matter density, FA, MD, ICVF, IsoVF, OD, QSM of VP, and FC in the VP and other brain regions among the PD‐LID, PD‐nLID and HC groups. In addition, the correlation between neuroimaging characteristics and UDysRS scores was analysed with partial correlation using age and disease duration as covariates. Significance was set at *p* < 0.05 within false discovery rate (FDR) correction (to counter the potential bias in multiple comparisons).

## RESULTS

### Demographic and clinical characteristics

The demographic and clinical characteristics of all participants are shown in Table 1. There were no significant differences in gender, age, or MoCA score among the three groups. Moreover, no significant differences were detected in disease duration, age of onset, H&Y stage, MDS‐UPDRS Part III scores, proportion of more affected body side, response to levodopa, or LEDD between the PD‐LID and PD‐nLID groups.

**TABLE 1:** Overview of clinical and demographic characteristics.

	PD‐LID	PD‐nLID	HC	*p* Values			
	(*n* = 31)	(*n* = 39)	(*n* = 28)	LID vs. nLID	LID vs. HC	nLID vs. HC	LID vs. nLID vs. HC
Gender: female/Male	18/13	25/14	19/9	NA	NA	NA	0.731^a^
Age, years	65.77 ± 8.66	65.38 ± 7.10	61.61 ± 5.27	NA	NA	NA	0.052^b^
Disease duration, years	9 (5–13)	7 (4–10)	NA	0.068^d^	NA	NA	NA
Age at onset, years	56.52 ± 8.49	58.51 ± 6.53	NA	0.270^c^	NA	NA	NA
MoCA score	21.10 ± 5.14	22.64 ± 3.48	22.82 ± 4.11	NA	NA	NA	0.215^b^
MDS‐UPDRS Part III score, ‘off’	38.68 ± 14.52	38.62 ± 13.73	NA	0.985^c^	NA	NA	NA
MDS‐UPDRS Part III score, ‘on’	23.23 ± 9.95	22.92 ± 9.45	NA	0.897^c^	NA	NA	NA
Response to levodopa, %	41.64 ± 14.0	39.94 ± 11.70	NA	0.583^c^	NA	NA	NA
More affected body side: left/right	17/14	20/19	NA	0.767^a^	NA	NA	NA
LEDD (mg)	979.44 ± 266.96	838.52 ± 344.75	NA	0.065^c^	NA	NA	NA
H&Y stage	3 (2.5–3)	3 (2.5–3)	NA	0.671^d^	NA	NA	NA
UDysRS score	32.65 ± 3.02	NA	NA	NA	NA	NA	NA

Abbreviations: H&Y stage: Hoehn and Yahr stage; HC: healthy control; LEDD: Levodopa Equivalent Daily Dose; MDS‐UPDRS Part III Movement Disorder Society‐Unified Parkinson's Disease Rating Scale; MoCA: Montreal Cognitive Assessment; NA: not applicable; UDysRS: Unified Dyskinesia Rating Scale.

^a^ Chi‐square test.

^b^ One‐way analysis of variance.

^c^ Two‐sample *t*‐test.

^d^ Mann–Whitney test.

### Neuroimaging group‐wise comparisons

#### Grey matter density

Analysis of variance showed no significant group differences in the grey matter density of the VP on the right side (*p* = 0.061). However, the grey matter density in the right VP was significantly lower in the PD‐nLID (*p* = 0.032) group than in the HC group (details in Appendix S1, Table S1).

#### Diffusion data analysis

**FIGURE 2:**
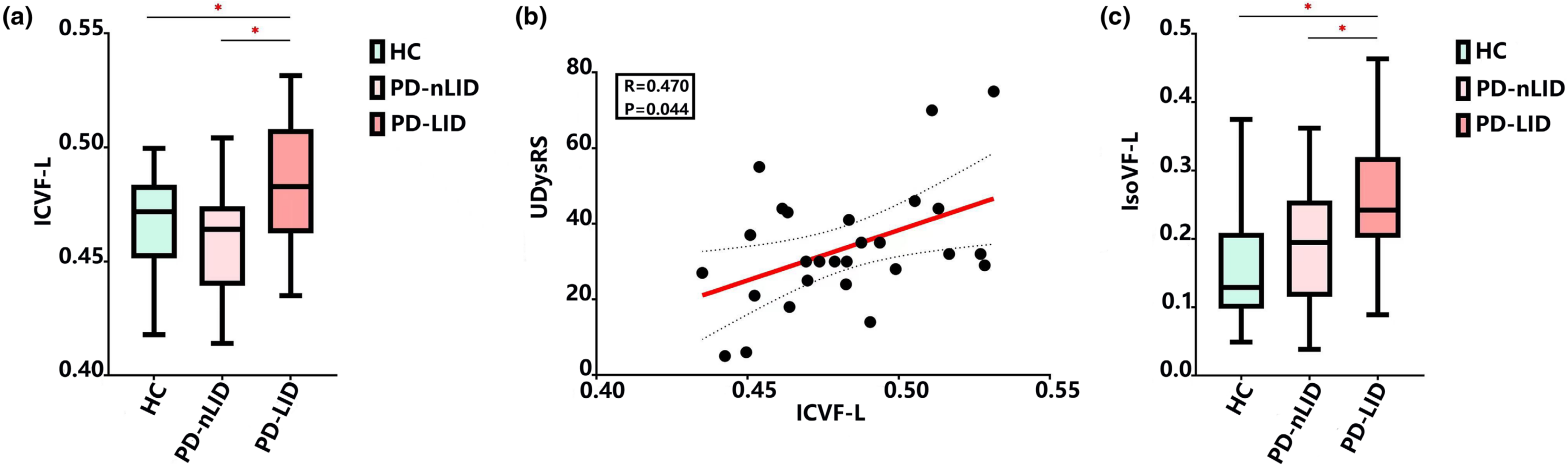
Comparison of intracellular volume fraction (ICVF) and isotropic volume fraction (IsoVF) values in the ventral pallidum (VP). (a) The ICVF values of the left VP in Parkinson's disease with levodopa‐induced dyskinesia (PD‐LID) were significantly higher than in Parkinson's disease without levodopa‐induced dyskinesia (PD‐nLID) and healthy controls (HCs). (b) The ICVF values positively correlated with Unified Dyskinesia Rating Scale (UDysRS) total scores. (c) The IsoVF values of the left VP in PD‐LID were significantly higher than in PD‐nLID and HC.

Analysis of variance showed significant group difference in ICVF (*p* = 0.001) and IsoVF (*p* = 0.021) of the left VP. The left VP had significantly higher ICVF and IsoVF in the PD‐LID group than in the PD‐nLID group (*p* = 0.023 and *p* = 0.028, respectively) and the HC group (*p* = 0.040 and *p* = 0.010, respectively; Figure 2a,c). ANOVA revealed no significant difference in the ICVF or IsoVF values of the right VP among the three groups (*p* = 0.587 and *p* = 0.118). In addition, the ICVF values of the left VP were positively associated with UDysRS total scores in PD‐LID patients (*R* = 0.470, *p* = 0.044; Figure 2b), but no significant correlation was found between the IsoVF of the left VP and UDysRS total scores (*R* = 0.083, *p* = 0.682). In addition, compared with the HC group, the MD in the right VP was significantly higher in the PD‐LID group (*p* = 0.023), and a trend for increased MD was observed in the PD‐nLID group (*p* = 0.067). However, ANOVA showed no significant differences in the MD on the left VP among the three groups (*p* = 0.153). Similarly, no significant differences in FA were observed on the two sides of the VP among the three groups (left: *p* = 0.959; right: *p* = 0.662; details in Appendix S1, Table S1).

#### Functional connectivity analysis

**FIGURE 3:**
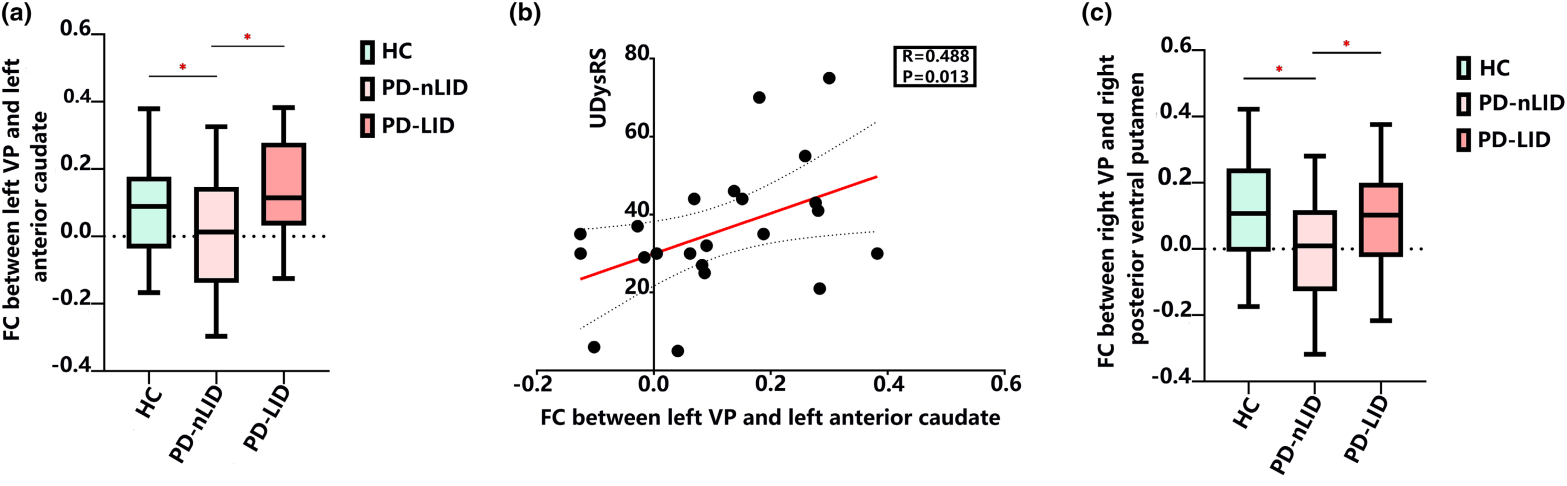
Comparison of functional connectivity (FC) between the ventral pallidum (VP) and other brain regions. (a) The Parkinson's disease without levodopa‐induced dyskinesia (PD‐nLID) group showed lower FC between the left VP and the left anterior caudate than the Parkinson's disease with levodopa‐induced dyskinesia (PD‐LID) and healthy control (HC) groups. (b) The FC changes between the left VP and the left anterior caudate positively correlated with Unified Dyskinesia Rating Scale (UDysRS) total scores. (c) The PD‐nLID group showed lower FC between the right VP and the right posterior ventral putamen than the PD‐LID and HC groups.

We analysed the FC between the bilateral VP and the brain regions in both hemispheres and found positive results. In the medication ‘off’ state, fMRI analysis revealed that the PD‐nLID group had significantly lower FC between the left VP and the left anterior caudate (Figure 3a), left middle frontal gyrus, and left precentral gyrus, as compared to the PD‐LID group (*p* = 0.014, *p* = 0.031 and *p* = 0.044, respectively) and the HC group (*p* = 0.001, *p* = 0.047 and *p* = 0.001, respectively). We also observed that the PD‐nLID group had lower FC between the right VP and right posterior ventral putamen (Figure 3c) and the right mediodorsal thalamus, as compared to the PD‐LID (*p* = 0.009 and *p* = 0.031, respectively) and the HC groups (*p* = 0.002 and *p* = 0.047, respectively). In the PD‐LID group, the FC between the left VP and left anterior caudate (*R* = 0.488, *p* = 0.013; Figure 3b) and between the left VP and left middle frontal gyrus (*R* = 0.433, *p* = 0.050) positively correlated with UDysRS total scores (details in Appendix S1, Table S2).

#### Quantitative susceptibility mapping analysis

Analysis of variance showed no significant difference in QSM values for the two sides of the VP among the three groups (left: *p* = 0.808; right: *p* = 0.418; details in Appendix S1, Table S1).

#### Relationships among different MRI parameters of interest

We separately assessed the relationships among MRI parameters that showed significant differences in the PD‐LID and PD‐nLID groups. After FDR correction, there was no significant correlation between different MRI parameters (details in Appendix S1).

## DISCUSSION

In the present study, we observed that ICVF and IsoVF values in the left VP in our PD‐LID group were significantly higher than those in the PD‐nLID and HC groups. The ICVF values in the PD‐LID group were positively correlated with the clinical severity of dyskinesia. In addition, in the medication ‘off’ state, the PD‐LID group exhibited a higher FC between the VP and putamen, caudate, mediodorsal thalamus and frontal gyrus than the PD‐nLID group. However, no significant difference in QSM values was found between these two PD groups, suggesting that there may be no significant metal deposition in the VP of PD‐LID patients. These results provide novel insights into the understanding of the potential role of VP underlying PD‐LID.

Multi‐shell dMRI with the NODDI model detected significantly higher ICVF and IsoVF values for the VP in the PD‐LID group than in the PD‐nLID and HC groups. The ICVF values were positively correlated with UDysRS total scores. ICVF refers to the space bounded by the membrane of neurites including axons and dendrites [33]. The higher ICVF in PD‐LID could be interpreted as an increase in synaptogenesis and dendritic spine density [41], which would further increase the number of structurally mature synapses and enhance the efficacy of synaptic transmission of the motor circuit. Alternatively, the higher ICVF in PD‐LID may potentially be associated with alterations in metabolism or mitochondrial dysfunction, resulting in axonal swelling [42, 43]. Higher IsoVF is typically associated with an elevated level of extracellular free water, which may be reflective of inflammation or neurodegeneration of the VP in PD‐LID [44]. In addition, the VP in the PD‐LID group showed higher MD than in the HC group in the DTI model, further reflecting the microscopic barrier disruption and accumulation of extracellular fluid accumulation in VP neurons [45].

The QSM values of the left VP positively correlated with ICVF values of the left VP in the PD‐LID group before FDR correction. We speculate that this may reflect compensatory structural alterations of the VP (i.e., the higher ICVF) due to the severe damage in dopaminergic neurons, although there was no significant correlation between different MRI parameters after FDR correction. Therefore, the specific mechanism of dyskinesia needs to be further studied.

Functional analysis in the medication ‘off’ state revealed that the VP in the PD‐LID group had higher FC with an extensive number of regions, including the striatum (i.e., anterior caudate and posterior ventral putamen), mediodorsal thalamus and cortical motor regions (i.e., the middle frontal gyrus and the precentral gyrus), in comparison to the PD‐nLID group. The FC between the VP and the anterior caudate as well as the middle frontal gyrus positively correlated with UDysRS total scores. Furthermore, no volume alterations of grey matter density were observed among the three groups, indicating that the altered FC patterns of the VP were not induced by anatomical changes. The altered FC suggests that the VP might regulate locomotion via the ventral striatum–VP–mediodorsal thalamus–cortex circuit in PD‐LID. Previous anatomical studies have demonstrated that GABAergic neurons in the VP receive inhibitory GABAergic input from ventral striatal regions and project inhibitory GABAergic efferents to the mediodorsal thalamus, which further projects excitatory glutamatergic efferents to the cortical motor regions [19, 46]. Furthermore, VP‐regulated motor dysfunction has been observed in animals with GABA receptor antagonist injected into the VP [8]. Previous studies have reported that the putamen mainly receives fibres from dopamine neurons and is most strongly affected by dopaminergic denervation in PD [47]. A PET study showed that PD‐LID patients receiving dopaminergic medications showed increased glutamatergic activity in the caudate, putamen and precentral gyrus compared with PD‐nLID patients. These findings suggest that dyskinetic patients may have abnormal glutamatergic transmission in motor areas following levodopa administration, which might be related to abnormal putamen signals [48]. Another study examining the topological properties of white matter demonstrated an increased node efficiency of the right putamen in dyskinetic PD patients, which may reflect the excessive transfer and processing of motor signals to other regions [49]. Combined with these findings, we speculate that alterations in both neuronal activation patterns and the information processing function of the VP, secondary to abnormal putamen signals, might contribute to the functional changes of the ventral striatum–VP–mediodorsal thalamus–cortex neural network. This, in turn, could exacerbate the over‐activation in the cortical motor regions and contribute to the emergence of dyskinesia.

In addition, we observed asymmetric motor symptoms and hemisphere differences in PD patients. LID usually has a unilateral onset [1]. A previous cohort study showed that PD patients with higher striatal asymmetric index had an increased susceptibility to dyskinesia [50]. Another study revealed that interhemispheric functional incoordination was correlated with the severity of dyskinesias in PD [51]. Some risk factors, including genetic and environmental factors, and aging could plausibly trigger such hemispheric differences [52]. Thus, we suspect that these imaging alterations might underlie the neural mechanisms of hemispheric asymmetries in PD patients with dyskinesia, which needs to be further investigated.

This study has some limitations. First, the sample size was relatively small; larger cohorts will be further recruited to validate the results. Second, as only cross‐sectional analyses were performed, longitudinal prospective evaluation of the VP in PD‐LID is needed to examine the causal relationship between these neuroimaging metrics and the development of PD‐LID. In addition, the FC changes before and after levodopa intake need to be investigated in the future.

In conclusion, the microstructural alterations of the VP (i.e., higher ICVF and IsoVF) were related to PD‐LID. Moreover, the connectivity changes between the VP and other brain regions (i.e., striatum, mediodorsal thalamus, and frontal lobe) revealed a clinical association of functional change in the ventral striatum–VP–mediodorsal thalamus–cortex network in PD‐LID. Our findings provide evidence of a role for microstructural alterations of VP and functional alterations of the VP‐mediated network in the pathological mechanism of PD‐LID.

## AUTHOR CONTRIBUTIONS

**Yawen Gan:** Data curation; writing – original draft; formal analysis. **Dongning Su:** Data curation; formal analysis; writing – original draft. **Zhe Zhang:** Software; writing – original draft; data curation. **Zhijin Zhang:** Data curation; conceptualization. **Rui Yan:** Formal analysis. **Zhu Liu:** Investigation. **Zhan Wang:** Methodology. **Junhong Zhou:** Writing – review and editing. **Joyce S. T. Lam:** Writing – review and editing. **Tao Wu:** Writing – review and editing. **Jing Jing:** Writing – review and editing; conceptualization. **Tao Feng**: Writing – review and editing; conceptualization.

## FUNDING INFORMATION

This research was supported by grants from the Natural Science Foundation of China (82071422 and 82271459) and Beijing Municipal Natural Science Foundation (7212031).

## CONFLICT OF INTEREST STATEMENT

The authors disclose no potential conflicts of interest.

## ETHICS STATEMENT

The studies involving human participants were reviewed and approved by the Ethics Committee of Beijing Tiantan Hospital, Capital Medical University, Beijing, China. All participants provided written informed consent prior to their participation in this study.

## Supporting information


Appendix S1

## Data Availability

The data that support the findings of this study are available from the corresponding author upon reasonable request.
