# Supplementary material for: Microstructural and functional alterations of the ventral pallidum are associated with levodopa‐induced dyskinesia in Parkinson's disease
Source: Eur J Neurol. 2023 Nov 17;31(2):e16147. doi: 10.1111/ene.16147 (PMC11235694; doi:10.1111/ene.16147)
Supplement: Supplementary file 1 — Appendix S1 [file ENE-31-e16147-s001.doc]

**Supplementary materials**

**Supplementary Methods**

**MRI Data Acquisition**

The 3D T1-weighted images were acquired using sagittal magnetization-prepared rapid gradient echo acquisition (MPRAGE) with the following parameters: TE/TR/TI = 2.0/2000/880 ms, flip angle = 8°, voxel size (AP×RL×SI) = 1×1×1 mm^3^, FOV (AP×RL×SI) = 240×208×256 mm^3^, and acceleration factor = 2. A 3D axial multi-echo gradient-echo (GRE) sequence was used for QSM data acquisition [1] with the following parameters: TE1/ TE2/ TE3/ TE4/TR = 6.7/13.4/20.1/26.8/32.0 ms, flip angle = 18°, voxel size (AP×RL×SI) = 0.9×0.9×0.9mm^3^, FOV (AP×RL×SI) = 230×208×158mm^3^, and acceleration factor = 4. The multi-shell dMRI images were acquired using the Center for Magnetic Resonance Research (CMRR) multi-band sequence (version R016a) [2] with the following parameters: TE/TR = 70/2800 ms, flip angle (excitation/refocusing) = 78°/160°, voxel size (AP×RL×SI) = 2×2×2 mm^3^, FOV (AP×RL×SI) = 210×210×144 mm^3^, multi-band factor =3, diffusion encoding using 50 directions at b = 1000 s/mm^2^ and 50 directions at b = 2000 s/mm^2^, plus 8 (5 AP phase-encoded and 3 PA phase-encoded) additional b = 0 images. The fMRI images were acquired using the CMRR multi-band sequence (version R016a) with the following parameters: TE/TR = 39/735 ms, flip angle= 52°, voxel size (AP×RL×SI) = 2.4×2.4×2.4mm^3^, FOV (AP×RL×SI) = 210×210×154 mm^3^, multi-band factor = 8, measurements = 490, and participants were informed to keep their eyes open and rest during scanning.

**dMRI analysis**

Field inhomogeneity-induced distortion, patient motion, and eddy current-related artifacts were corrected using the TOPUP and EDDY toolbox[3] in FSL. In detail, AP phase-encoded and PA phase-encoded b = 0 images were used to generate the field inhomogeneity map. The field inhomogeneity information and all AP-encoded images (50 directions at b = 1000 s/mm^2^, 50 directions at b = 2000 s/mm^2^, plus 5 b = 0) were used in EDDY toolbox to correct the image artifacts. The diffusion images after correction were then used for diffusion tensor imaging (DTI, using only the b=1000 s/mm^2^ shell data with b = 0) and NODDI[4] (using two-shell data of b = 1000 s/mm^2^ , and b = 2000 s/mm^2^ with b = 0) model fitting. The NODDI model fitting used convex optimization for acceleration with AMICO toolbox[5].

**fMRI imaging analysis**

Preprocessing of rs-fMRI images included removal of the first 10 volumes; realignment; slice-time correction; segmentation of grey matter, white matter, and CSF; normalization to the MNI template; and spatial smoothing based on a Gaussian kernel set at 6-mm full width at half-maximum. Nuisance variable regression was then performed, and the first five principal components from the segmented white matter and CSF were regressed out of the signal.

The six motion realignment parameters and their first-order derivatives and outlier volumes detected in the scrubbing procedure were similarly regressed out of the signal. We discarded acquisitions if they had mean framewise displacement values >0.2 mm or if the maximum displacement was greater than one voxel size (2 mm). According to these criteria, we excluded three participants consisting of two PD-LID and one PD-nLID. The data were then linearly detrended, and the residual signals were bandpass filtered at 0.01 to 0.08 Hz.

**Supplementary Results**

**The relationships between different MRI parameters of interest**

The correlation between different MRI parameters was analyzed with partial correlation using age and disease duration as covariates and corrected by FDR with the significance levels at *P* < 0.05. After FDR correction, there was no significant correlation between different MRI parameters. Before FDR correction, the QSM values of left VP positively correlated with the ICVF values of left VP in PD-LID (R = 0.405，*P* = 0.036). However, no significant correlation was found between the QSM values and ICVF values of the left VP in PD-nLID (R = -0.034，*P* = 0.855).

**Paired t test of clinical and imaging variables of both sides in two PD groups**

We compared the clinical symptoms (PD-nLID: right 13.15 ± 0.98, left 11.10 ± 5.32, *P* = 0.082; PD-LID: right 12.84 ± 1.14, left 11.10 ± 0.99, *P* = 0.076) and the imaging variables of both sides with paired t-tests (In the PD-LID group: QSM right 0.034 ± 0.020, left 0.018 ± 0.014, *P* = 0.000; ICVF right 0.522 ± 0.041, left 0.484 ± 0.028, *P* = 0.000; IsovF right 0.145 ± 0.060, left 0.244 ± 0.101, *P* = 0.000; FA right 0.273±0.044, left 0.219 ± 0.022, *P* = 0.000; MD right 0.0008 ±0.00006, left 0.0008 ± 00007, *P* = 0.000; Grey matter density *P* =0.932; FC between VP and anterior caudate,middle frontal gyrus, precentral gyrus, posterior ventral putamen, mediodorsal thalamus *P* = 0.106, *P* = 0.096, *P* = 0.490, *P* = 0.132, *P* = 0.155; In the PD-nLID group: QSM right 0.033 ± 0.02, left 0.016 ± 0.015, *P* = 0.001; ICVF right 0.517 ± 0.053, left 0.473 ± 0.045, *P* = 0.009; IsovF *P* = 0.792; FA right 0.268 ± 0.058, left 0.223 ± 0.091, *P* = 0.001; MD right 0.0008 ± 0.00005, left 0.0008 ± 00009, *P* = 0.038; Grey matter density right 0.352 ± 0.031, left 0.356 ± 0.034, *P* = 0.007; FC between VP and anterior caudate,middle frontal gyrus, precentral gyrus, posterior ventral putamen, mediodorsal thalamus *P* = 0.972, *P* = 0.916, *P* = 0.873, *P* = 0.724, *P* = 0.675).

TABLE S1. One-way ANOVA of grey matter density, FA, MD, ICVF, IsoVF, OD, QSM of VP among the three groups

| Imaging parameters | *P* values | | | |
| --- | --- | --- | --- | --- |
|  | LID vs nLID | LID vs HC | nLID vs HC | LID vs nLID vs HC |
| Grey matter density (left) | NA | NA | NA | 0.625 |
| Grey matter density (right) | NA | NA | 0.032^*^ | 0.061 |
| ICVF (left) | 0.023^*^ | 0.040^*^ | 0.305 | 0.001^*^ |
| ICVF (right) | NA | NA | NA | 0.587 |
| IsovF (left) | 0.028^*^ | 0.010^*^ | 0.543 | 0.021^*^ |
| IsovF (right) | NA | NA | NA | 0.118 |
| FA (left) | NA | NA | NA | 0.959 |
| FA (right) | NA | NA | NA | 0.662 |
| MD (left) | NA | NA | NA | 0.153 |
| MD (right) | 0.498 | 0.023^*^ | 0.067 | 0.047^*^ |
| OD (left) | NA | NA | NA | 0.930 |
| OD (right) | NA | NA | NA | 0.596 |
| QSM (left) | NA | NA | NA | 0.808 |
| QSM (right) | NA | NA | NA | 0.418 |

**P* < 0.05 was set significant.

TABLE S2. One-way ANOVA of functional connectivity among the three groups

| Seed | Brain regions | P values | | | |
| --- | --- | --- | --- | --- | --- |
|  |  | LID vs nLID | LID vs HC | nLID vs HC | LID vs nLID vs HC |
| Left VP | left anterior caudate | 0.014^*^ | 0.311 | 0.001^*^ | 0.002^*^ |
|  | left middle frontal gyrus | 0.031^*^ | 0.257 | 0.047^*^ | 0.010^*^ |
|  | left precentral gyrus | 0.044^*^ | 0.136 | 0.001^*^ | 0.002^*^ |
| Right VP | right posterior ventral putamen | 0.009^*^ | 0.734 | 0.002^*^ | 0.003^*^ |
|  | right mediodorsal thalamus | 0.031^*^ | 0.082 | 0.047^*^ | 0.036^*^ |

**P* < 0.05 was set significant.

**Reference**

1. Liu T, Xu W, Spincemaille P, Avestimehr AS, Wang Y. Accuracy of the morphology enabled dipole inversion (MEDI) algorithm for quantitative susceptibility mapping in MRI. *IEEE Trans Med Imaging*. 2012;**31:**816-824.

2. Xu J, Moeller S, Auerbach EJ*, et al.* Evaluation of slice accelerations using multiband echo planar imaging at 3 T. *Neuroimage*. 2013;**83:**991-1001.

3. Andersson JLR, Sotiropoulos SN. An integrated approach to correction for off-resonance effects and subject movement in diffusion MR imaging. *Neuroimage*. 2016;**125:**1063-1078.

4. Zhang H, Schneider T, Wheeler-Kingshott CA, Alexander DC. NODDI: practical in vivo neurite orientation dispersion and density imaging of the human brain. *Neuroimage*. 2012;**61:**1000-1016.

5. Daducci A, Canales-Rodríguez EJ, Zhang H, Dyrby TB, Alexander DC, Thiran JP. Accelerated Microstructure Imaging via Convex Optimization (AMICO) from diffusion MRI data. *Neuroimage*. 2015;**105:**32-44.
